# Instructions in Nitrous Oxide

**Published:** 1866-10

**Authors:** 


### INSTRUCTIONS IN NITROUS OXIDE.

By Geo. T. Barker, D. D. S., *Professor of the Principles of Dental Surgery and Therapeutics, in the Pennsylvania College of Dental Surgery, etc., etc.*

This is a neat, large-type, well gotten up little book of sixty-one pages (just the orthodox size), published by that well known firm Rubencame & Stockton. It will be found a good thing for the student; and nearly all the profession are but students, as far as the protoxyd of nitrogen is concerned.

The book can be had at the dental depots.

